# Discovery of Small Molecules That Target Vascular Endothelial Growth Factor Receptor-2 Signalling Pathway Employing Molecular Modelling Studies

**DOI:** 10.3390/cells8030269

**Published:** 2019-03-21

**Authors:** Shailima Rampogu, Ayoung Baek, Chanin Park, Minky Son, Shraddha Parate, Saravanan Parameswaran, Yohan Park, Baji Shaik, Ju Hyun Kim, Seok Ju Park, Keun Woo Lee

**Affiliations:** 1 Division of Life Sciences, Division of Applied Life Science (BK21 Plus), Plant Molecular Biology and Biotechnology Research Center (PMBBRC), Research Institute of Natural Science (RINS), Gyeongsang National University (GNU), 501 Jinju-daero, Jinju 52828, Korea; shailima.rampogu@gmail.com (S.R.); ayoung@gnu.ac.kr (A.B.); chaninpark0806@gmail.com (C.P.); minky@gnu.ac.kr (M.S.); parateshraddha@gmail.com (S.P.); dr.p.saravanan.bi@gmail.com (S.P.); 2 College of Pharmacy, Inje University, 197 Inje-ro, Gimhae, Gyeongnam 50834, Korea; yohanpark@inje.ac.kr; 3 Department of Chemistry (BK 21 plus), Research Institute of Natural Science (RINS), Gyeongsang National University, Jinju, Gyeongnam 52828, Korea; shaikbaji2@gmail.com (B.S.); juhyun@gnu.ac.kr (J.H.K.); 4 Department of Internal Medicine, College of Medicine, Busan Paik Hospital, Inje University, Gyeongnam 47392, Korea

**Keywords:** anti-angiogenic inhibitors, type-II anti-angiogenic inhibitors, VEGFR-2, natural products, protein kinase inhibitors

## Abstract

Angiogenesis is defined as the formation of new blood vessels and is a key phenomenon manifested in a host of cancers during which tyrosine kinases play a crucial role. Vascular endothelial growth factor receptor-2 (VEGFR-2) is pivotal in cancer angiogenesis, which warrants the urgency of discovering new anti-angiogenic inhibitors that target the signalling pathways. To obtain this objective, a structure-based pharmacophore model was built from the drug target VEGFR-2 (PDB code: 4AG8), complexed with axitinib and was subsequently validated and employed as a 3D query to retrieve the candidate compounds with the key inhibitory features. The model was escalated to molecular docking studies resulting in seven candidate compounds. The molecular docking studies revealed that the seven compounds displayed a higher dock score than the reference-cocrystallised compound. The GROningen MAchine for Chemical Simulations (GROMACS) package guided molecular dynamics (MD) results determined their binding mode and affirmed stable root mean square deviation. Furthermore, these compounds have preserved their key interactions with the residues Glu885, Glu917, Cys919 and Asp1046. The obtained findings deem that the seven compounds could act as novel anti-angiogenic inhibitors and may further assist as the prototype in designing and developing new inhibitors.

## 1. Introduction

Cancer development is demonstrated by a characteristic uncontrolled growth of cells leading to enormous biological variation [1]. Angiogenesis is a process of formation of new blood vessels that predominantly display a fundamental role in cancer development and metastasis [2,3,4]. Amplification of angiogenic factors are closely related to the tumour angiogenesis, which induces remarkable morphological changes to the tumour vessels and extracellular matrix proteins located on cell surface, thereby, serving as markers in differentiating normal cells from abnormal cells [2]. The research targeting angiogenic factors as a strategy to ameliorate cancer metastasis was increased after Folkman’s postulates that highlighted the importance of angiogenesis in tumour growth [5,6].

Vascular endothelial growth factor (VEGF) inhibitors are extensively studied to combat metastasis, and several inhibitors have been discovered [1,7,8]. VEGFs comprises of VEGF-A, VEGF-B, VEGF-C, VEGF-D and placental growth factor (PLGF) [9,10] and function by triggering cellular responses after binding to the VEGF receptors (VEGFR) located on the plasma membrane [9]. The VEGFR consists of VEGFR-1, VEGFR-2 and VEGFR-3 which regulate the angiogenic effect of VEGF ligands [1,11]. Amongst them, VEGF-A is vital in rendering the tumour angiogenesis signals through VEGFR-2. VEGFR-2 is a major VEGF-A signalling receptor [6]. Strategically impeding the signals of tyrosine kinase VEGFR-2 signalling pathway could distort the angiogenesis process, thereby, hindering and alleviating the proliferation of tumour [12,13,14]. 

The tyrosine kinase inhibitors are broadly divided into type I and type II based on their binding motif [10]. The type I inhibitors bind to the front ATP binding pocket observed in active enzymatic form demonstrating a Asp-Phe-Gly (DFG)-in conformation, while the type II inhibitors bind to both the ATP binding site and the hydrophobic back pocket noticed in the inactive form of kinase, represented by DFG-out conformation [15]. The front pocket consists of two key residues Glu917 and Cys919 and the back pocket contains Glu885 and Asp1046 residues. Hydrogen bond interactions with these residues is critically essential, the back-to-front approach of binding pattern is proven to be a highly efficient strategy in kinase drug development pipeline [15,16,17,18]. 

In the recent times, several anti-angiogenic inhibitors have been discovered and a few have obtained the FDA approval as well [19]. However, the tumours demonstrate resistance by evading these inhibitors and spread [20], thereby, exhibiting remarkable toxicities, such as profuse bleeding, hypertension and fatigue [1,21]. This condition puts forth the demand for new inhibitors with greater therapeutic potential. Natural products (NPs) have contributed immensely towards the drug development process exhibiting enormous medicinal values [22]. Additionally, a variety of NPs have displayed anticancer properties due to their diverse scaffolds, specificity, target affinity, low side effects and their abundance [23,24,25,26,27,28]. Predominantly, NPs, such as plant extracts or the phytochemicals, are widely popular amongst researchers due to their pleiotropic nature in function [29]. Furthermore, it is evidenced that the Asian population demonstrate a relatively reduced risk of cancer genesis in comparison to the West as the diet of Asians includes more vegetables and fruits [30,31]. The bioactive compounds present in these diets regulate the signalling pathways associated with cancer [30,32,33]. Although reports exist on the use of phytochemicals as anti-angiogenic [30,34,35], there is still an unexplored avenue of phytochemicals with potential anti-angiogenic ability. 

Encouraged by these reports, the current investigation aims to identify the potential phytochemicals that demonstrate a back-to-front binding mechanism employing pharmacophore-modelling studies. 

## 2. Results

### 2.1. Structure-Based Pharmacophore Generation

Structure-based pharmacophore was generated from human VEGFR-2 kinase domain (PDB code: 4AG8) in complex with axitinib. Axitinib is an approved drug, and its binding mode along with its key features have been exploited in the generation of the pharmacophore model. Furthermore, reports exist that the back-to-front approach in binding renders efficient inhibitory activity [15]. Correspondingly, a total of ten pharmacophore models have been generated with varied features, Table 1. The best model was selected based on the selectivity score and the features that are in harmony with the key residues.

**Figure 1:**
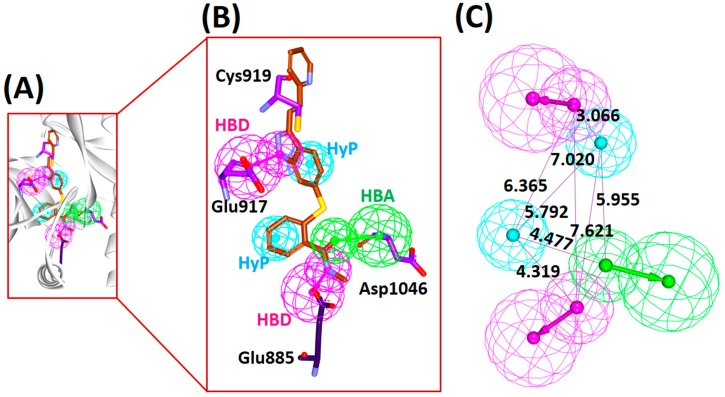
Structure-based pharmacophore model generation. (**A**) Generated pharmacophore model at proteins active site. (**B**) Pharmacophore features complementary to the key residues. (**C**) Interfeature distance between the pharmacophore features. HBA, hydrogen bond acceptor; HBD, hydrogen bond donor; HyP, hydrophobic.

Accordingly, a five-featured pharmacophore (Model 1) was chosen that demonstrated the highest selectivity score of 9.84. Upon scrupulous examination for the key features, it was revealed that the model comprised of two hydrogen bond donors (HBD), two hydrophobic features (HyP) and one hydrogen bond acceptor (HBA) feature complemented by the key residues, Glu917-Cys919 at the front pocket and Glu885-Asp1046 towards the back pocket, Figure 1.

### 2.2. Validation of the Selected Pharmacophore Model

The chosen pharmacophore model was assessed for its efficacy in distinguishing between active and inactive compounds, thereby, retrieving the active compounds. In this pursuit, the model was validated by two different methods, such as the Receiver Operating Characteristic (ROC) curve and the Güner-Henry, also referred to as the decoy set method. 

**Figure 2:**
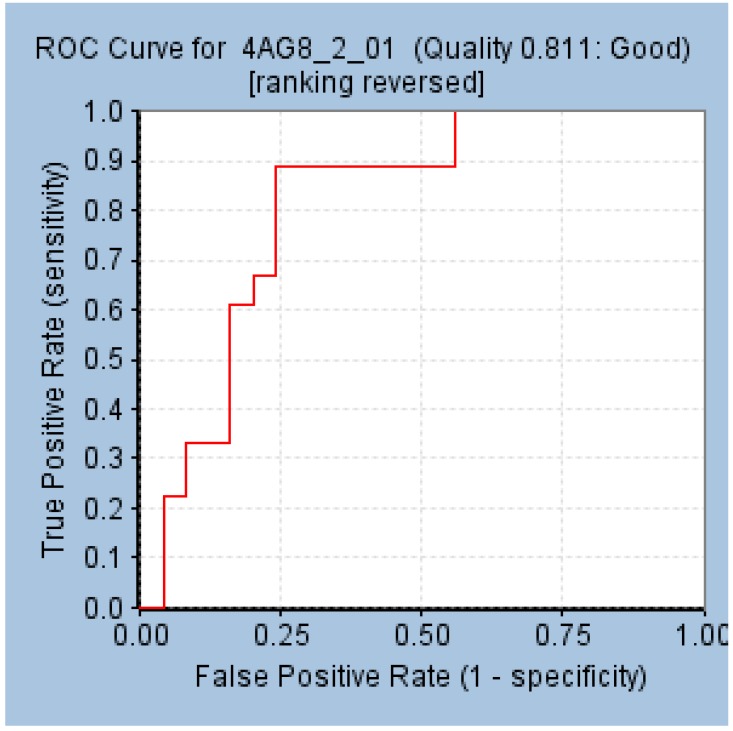
Validation of the selected pharmacophore model by receiver operating characteristic (ROC) curve.

The results generated by ROC demonstrated that the model has aligned to all the 18 compounds and three inactive compounds. Furthermore, the area under the curve (AUC) was elucidated to be 0.81 illuminating the model to be a good model, Figure 2. The decoy set method of validation has resulted in retrieving 26 compounds (Ht) with 23 active compounds (Ha) in it demonstrating 95.8% active compounds. The corresponding goodness of fit (GF) score was computed as 0.87 endorsing the potential of the model, Table 2. Both the validations have ensured the superiority of the pharmacophore model in retrieving active compounds from a given database.

### 2.3. Virtual Screening for the Retrieval of Drug-Like Compounds

**Figure 3:**
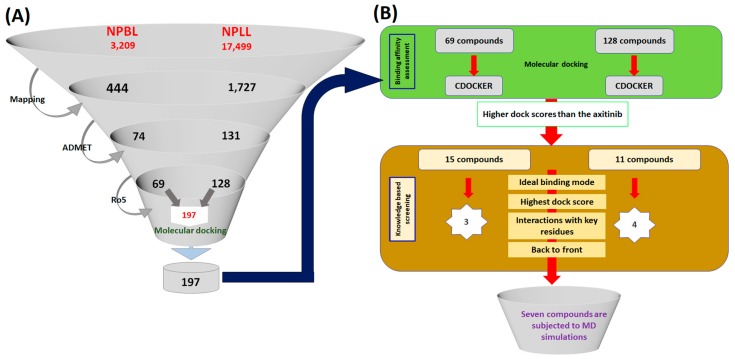
Pictorial depiction of the steps involved in the identification of the potential compounds. (**A**) Illustration of virtual screening and drug-like assessment process. (**B**) Molecular docking guided retrieval of the prospective compounds.

The well-validated pharmacophore model was used as the query to screen the natural compounds available from Eximed (https://eximedlab.com/). This consists of compounds from the known and the already available natural products named as Natural-Product-Based Library (NPBL) and Natural-Product-Like Library (NPLL) that are similar to the natural compounds. This library comprises compounds designed from the combinatorial synthesis of natural compounds. The ligand pharmacophore mapping has retrieved 444 compounds from the NPBL and 1727 compounds from NPLL, respectively. Upon subsequent application of the absorption, distribution, metabolism and excretion–toxicity (ADMET) filters and the Lipinski’s Rule of 5, a total of 197 compounds, Figure 3A, have been resulted that were subsequently upgraded to molecular docking studies.

### 2.4. Molecular Docking-Based Screening

Molecular docking delineates on the binding affinities between the target proteins and the virtually screened ligands. Furthermore, the molecular docking findings elucidates binding modes of the ligands. In the current study, the 197 virtually screened ligands were upgraded to CDOCKER docking protocol available on the Discovery Studio v4.5 (DS). The cocrystallised axitinib that was used as the reference demonstrated a -Cdocker interaction energy of 49.22 kcal/mol. Correspondingly, from the largest cluster, 15 and 11 compounds from NPBL and NPLL, respectively, generated a higher dock score than the reference and were labelled as Hits, Figure 3B. These compounds were scrupulously analysed for their interactions with the key residues Glu885, Cys919, Glu917 and Asp1046 complemented by the back to front approach that resulted in three and four compounds from the NPBL and NPLL, respectively. These seven compounds were escalated to molecular dynamics (MD) simulations to affirm the molecular docking results and to evaluate the stability of the protein-ligand complex system.

### 2.5. Molecular Dynamics Simulations Guided Binding Mode Analysis

To additionally gain insight into the dynamic behaviour of the small molecules at the active site of the protein and to further, affirm the molecular docking results the MD simulations were executed for 50 ns employing GROningen MAchine for Chemical Simulations (GROMACS) package. The obtained results were read as the root mean square deviation (RMSD). 

### 2.6. Elucidation of Protein Stability and Binding Mode Analysis

**Figure 4:**
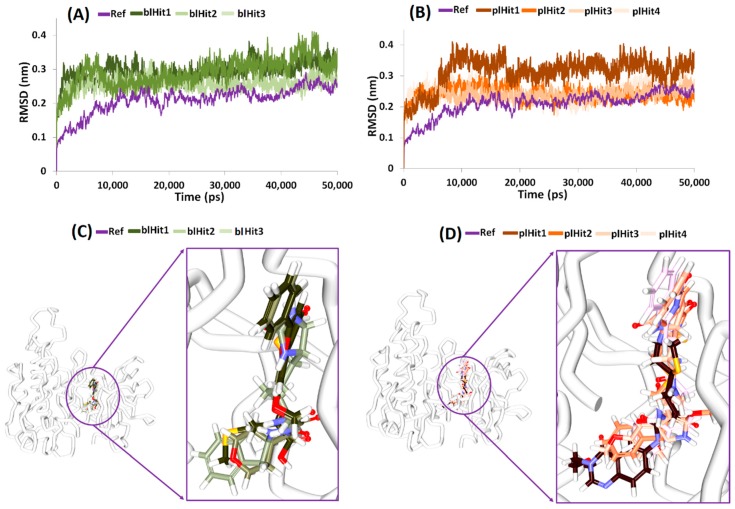
Results obtained through molecular dynamics (MD) studies. (**A**) Root mean square deviation (RMSD) of the protein-hit compounds from the Natural-Product-Based Library (NPBL) database. (**B**) RMSD of the protein-hit compounds from the Natural-Product-Like Library (NPLL) database. (**C**) Binding mode analysis of compounds from NPBL database. (**D**) Binding mode analysis of compounds from NPLL database.

The RMSD profiles of the chosen compounds from NPBL and NPLL were observed to be stable and in agreement with the reference compound. Furthermore, all the compounds generated an RMSD below 0.4 nm, Figure 4A,B. To further elucidate on the binding modes of the compounds, the representative structures from last 10 ns were extracted and were subsequently superimposed. Upon vigilant analysis, it was perceived that the compounds were similarly positioned at the binding pocket as the cocrystal (also the reference compound), aided by several charged residues, Figure 4C,D.

### 2.7. Deciphering on Intermolecular Interactions 

#### 2.7.1. Protein-Reference 

**Figure 5:**
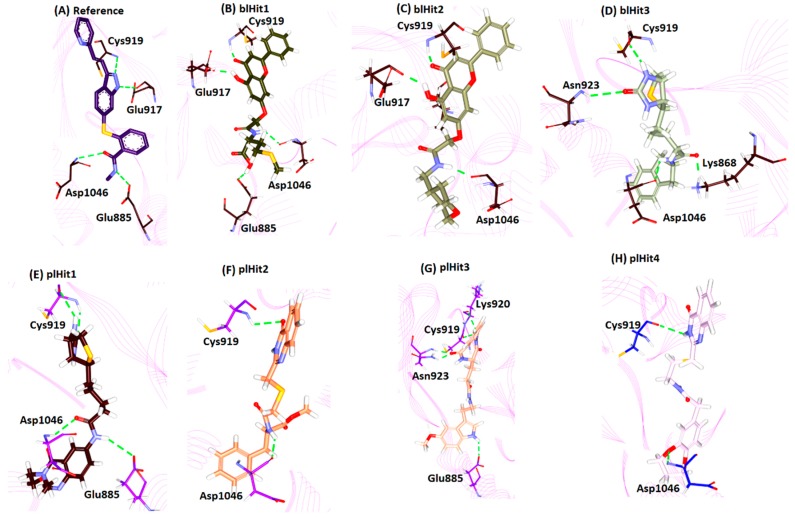
Intermolecular hydrogen bond interaction of the protein-hit compounds in comparison with the reference. (**A**) Intermolecular hydrogen bond interactions of the reference compound; (**B**) Hydrogen bond interactions of the blHit1; (**C**) Hydrogen bond interactions of the blHit2; (**D**) Hydrogen bond interactions of the blHit3; (**E**) Hydrogen bond interactions of the plHit1; (**F**) Hydrogen bond interactions of the plHit2; (**G**) Hydrogen bond interactions of the plHit3; (**H**) Hydrogen bond interactions of the plHit4. Green dotted line represent the hydrogen bond interactions.

Further, upon elucidating on the intermolecular interactions of the reference compound, it was revealed that the reference had interacted with the key residues Glu885, Glu917, Cys919 and Asp1046 represented by hydrogen bonds with an acceptable bond length, Figure 5A and Table 3. The OE2 atom of Glu885 (H-acceptor) interacted with the N82 atom (H-donor) of the ligand with a bond length of 2.6 Å. The N atom of the residue Asp1046 (H-donor) interacted with the O81 (H-acceptor) of the ligand with a bond length of 2.9 Å. The O atom of the residue Glu917 (H-acceptor) and the N15 atom (H-donor) of the ligand interacted by a hydrogen bond with a bond length of 2.8 Å. Another hydrogen bond was formed between the N atom of Cys919 (H-donor) and N14 atom (H-acceptor) of the ligand with a bond length of 2.9 Å. Furthermore, ring A of the reference compound interacted with Val848 (π–alkyl, bond length 5.0 Å), Ala866 (π–alkyl, bond length 5.4 Å), Lys868 (π–cation, bond length 4.3 Å) and Val916 (π–σ, bond length 3.5 Å) residues, correspondingly. The ring B has interacted with Ala866 (π–alkyl, bond length 4.3 Å), Val848 (π–alkyl, bond length 4. 6 Å), Leu1035 (π–σ, bond length 3.5 Å), Cys1045 (π–alkyl, bond length 4.8 Å) and Phe1047 (π–π T shaped, bond length 4.8 Å) residues and ring C with Ala866 (π–alkyl, bond length 3.6 Å), and Leu1035 (π–σ bond length 3.7 Å), respectively. Additionally, the residues Val867, Leu889, Val899, Val914, Phe918, Lys920 and Gly922 aided the ligand to be positioned at the active site and ring D with Leu840 (π–alkyl, bond length 4.4 Å), Figure 5A and Table 3 and Supplementary Figure S1.

Moreover, three compounds from the NPBL database represented the key residue interactions and were labelled as blHit1, blHit2 and blHit3 to distinguish them from the NPLL which are labelled as pl.

#### 2.7.2. Protein-blHit1

The compound blHit1 rendered the interaction with the key residues from both the pockets including Glu885, Glu917, Cys919 and Asp1046, respectively with an acceptable bond length. Additionally, this compound interacted with the key residues from the front and back pocket. The OE2 atom of Glu885 (H-acceptor) and H40 atom (H-donor) of the ligand interacted by the Hydrogen bond interaction with a bond distance of 2.1 Å. The residue Asp1046, with its O atom (H-acceptor) interacted with H35 atom (H-donor) of the ligand projecting a bond length of 2.0 Å. The O atom (H-acceptor) of residue Glu917 interacted with H37 (H-donor) atom of the ligand with a bond length of 1.9 Å. The NH atom of Cys919 (H-donor) and the O15 atom (H-acceptor) of the ligand rendered a bond length of 1.9 Å. Moreover, residues, such as Leu840, Val848, Ala866, Leu1035 and Cys1045, firmly clamped the ligand by π–alkyl interactions. The ring A of the ligand interacted with Leu840 (π–alkyl, bond length 4.8 Å), ring B has joined to Leu840 (π–alkyl, bond length 5.0 Å), and Ala866 (π–alkyl, bond length 4.2 Å). Ring C has interacted with Val 848 (π–alkyl, bond length 4.9 Å), Ala866 (π–alkyl, bond length 4.1 Å), Leu1035 (π–alkyl, bond length 5.1 Å) and Cys1045 (π–alkyl, bond length 4.9 Å), respectively. The residues Ile888, Leu889, Ile892, Val899, Val914, Val916, Lys920, Gly922, Asn923, Thr926, His1026, Ile1044, Phe1047 and Ala1050 participated in van der Waals interactions clamping the ligand firmly at the active site, Figure 5B and Table 3 and Supplementary Figure S1.

#### 2.7.3. Protein-blHit2

The compound blHit2 was rendered by three hydrogen bonds with the residues Glu917, Cys919 and Asp1046, respectively. The O atom (H-acceptor) of the residue Glu917 interacted with the H35 atom (H-donor) of the ligand demonstrating a bond length of 1.9 Å. Another key residue Cys919 with its HN atom (H-donor) joined to the O12 (H-acceptor) atom of the ligand representing a bond length of 1.8 Å. The O atom (H-acceptor) of Asp1046 residue joined to the H34 atom of the ligand (H-donor) represented by a bond length of 1.9 Å. Furthermore, several residues interacted with the ligand for an appropriate positioning at the active site. The ring A of the ligand interacted with Leu889 (alkyl hydrophobic, bond length 4.9 Å) and the ring B joined with Ala866 (π–alkyl, bond length 4.2 Å), Val848 (π–alkyl, bond length 5.1 Å), Leu1035 (π–alkyl, bond length 4.8 Å) and Cys1045 (π–alkyl, bond length 4.8 Å), respectively. The ring C joined with Leu840 (π–alkyl, bond length 5.1 Å), Ala866 (π–alkyl, bond length 4.2 Å), Cys919 and Leu1035 and ring D interacted with Leu840, correspondingly. The residues Lys868, Glu885, Ile888, Ile892, Val899, Phe918, Lys920, Gly922, Val914, Asn923, Thr926, Ile1044, and Phe1047 held the ligands by van der Waals interactions, Figure 5C and Table 3 and Supplementary Figure S1.

#### 2.7.4. Protein-blHit3

The blHit3 formed four hydrogen bond interactions with the key residues Lys868, Cys919 Asn923, and Asp1046, respectively rendered by an allowable bond length. The HZ3 atom (H-donor) of Lys868 residue interacted with the O17 atom (H-acceptor) of the ligand by a bond length of 1.9 Å. Another hydrogen bond was formed between O atom of Cys919 (H-acceptor) and the H27 atom (H-donor) of the ligand representing an acceptable bond length of 2.1 Å. The HN atom (H-donor) of Asn923 interacted with the O14 atom (H-acceptor) of the ligand with an allowable bond length of 2.8 Å. The O atom (H-acceptor) of the residue Asp1046 and the H34 atom (H-donor) of the ligand formed a hydrogen bond with a length of 2.1 Å; Figure 5D and Table 3. The ring A of the ligand and His1026 interacted by π–π staked interactions with a bond length of 5.1 Å. Additionally, the residues, such as Val848, Ala866, Glu885, Ile888, Leu889, Ile892, Val899, Val916, Phe918, Gly922, Leu1019, Leu1035, Ile1044 and Phe1047, have favoured proper positioning of the ligand at the active site of the protein, Supplementary Figure S1.

#### 2.7.5. Protein-plHit1

The compounds retrieved from NPLL (plHit1, plHit2, plHit3 and plHit4) were also analysed for their intermolecular interactions. The compound plHit1 prompted four hydrogen bonds with the residues Glu885, Cys919 and Asp1046, correspondingly. The OE2 atom (H-acceptor) of the residue Glu885 interacted with H41 atom (H-donor) of the residue with a bond length of 2.8 Å. The O atom of Cys919 (H-acceptor) interacted with the H33 atom of the ligand (H-donor) demonstrated by a bond length of 2.6 Å. The HN atom of Cys919 (H-donor) interacted with the O19 of the ligand (H-acceptor) rendered by a bond length of 2.1 Å. The NH atom (H-donor) of Asp1046 demonstrated a hydrogen bond interaction with the O21 atom (H-acceptor) of the ligand by a bond length of 2.6 Å. Interestingly, the sulphur atom ring of B has interacted with the Leu840 residue by a sulphur-X type of bond demonstrated by a length of 3.2 Å. The ring C of the ligand and the key residue Asp1046 demonstrated a π–σ bond with a bond length of 2.7 Å. Furthermore, the ring D of the ligand interacted with His1026 residue by π–π stacked hydrophobic interaction with a bond length of 4.6 Å. The residues Gly841, Ala866, Leu889, His891, Val899, Val914, Val916, Glu917, Gly922, Asn923, Met1016, Leu1019, Arg1022, Cys1024, Ile1025, Arg1027, Leu1035, Cys1045, Phe1047 and Ala1050 assisted in positioning the ligand at the active site of the protein, Figure 5E and Table 3 and Supplementary Figure S1.

#### 2.7.6. Protein-plHit2

The plHit2 formed two hydrogen bond interactions with Cys919 and Asp1046, respectively. The HN atom (H-donor) of Cys919 and O11 atom (H-acceptor) of the ligand interacted via a hydrogen bond interaction with a length of 2.6 Å. Another hydrogen bond interaction was noticed between the O atom (H-acceptor) of Asp1046 and H32 atom (H-donor) of the ligand demonstrated by a distance of 2.0 Å. Furthermore, the ring A interacted with Leu889 (π–alkyl, bond length 5.3 Å), ring B interacted with Leu840 (π–alkyl, bond length 4.9 Å), Ala866 (π–alkyl, bond length 4.4 Å), and Cys919 (π–alkyl, bond length 5.4 Å) and ring C has interacted with Leu840 (π–σ, bond length 2.6 Å), respectively. Several other residues, such as Glu885, Ile892, Val899, Val914, Glu917, Phe918, Lys920, Gly922, Asn923, Leu1019, His1026, Leu1035, Ile1044 and Phe1047, clamped the ligand within the active site of the protein, Figure 5F and Table 3 and Supplementary Figure S1.

#### 2.7.7. Protein-plHit3

The plHit3 resulted in the highest number of hydrogen bonds with the key residues located at the active site, such as Glu885, Cys919, Lys920 and Asn923, respectively. The OE2 atom (H-acceptor) of Glu885 and the H34 atom (H-donor) of the ligand formed a hydrogen bond with a bond length of 2.2 Å. The O atom of the key residue Cys919 (H-acceptor) and H32 atom (H-donor) of the ligand joined with a bond length of 2.4 Å. Another hydrogen bond was noted between the O atom of Lys920 and the H32 atom of the ligand with a bond length of 2.9 Å. The residue Asn923 formed two hydrogen bonds with atom HD21 and HN with its corresponding ligand atom O14 rendered by a distance of 1.9 Å each, respectively. Moreover, several residues were noticed to clamp the ligand at the active site. The ring A interacted with Leu889 (π–alkyl, bond length 4.3 Å), ring B joined with Leu889 (π–σ, bond length 2.8 Å), Lys868 (π–cation, bond length 3.1 Å) and Val916 (π–alkyl, bond length 4.6 Å) and ring D interacted with Leu840 (π–cation, bond length 4.3 Å), respectively. Furthermore, the residues Leu840, Val848, Ala866, Ile888, Ile892, Val899, Val914, Glu917, Phe918, Gly922, His1026, Leu1035, Ile1044, Cys1045, Asp1046, Phe1047 and Leu1091 favoured proper seating of the ligand at the active site of the protein, Figure 5G and Table 3 and Supplementary Figure S1.

#### 2.7.8. Protein-plHit4

The compound plHit4 projected two hydrogen bonds represented by the key residues Cys919 and Asp1046, respectively. The O atom (H-acceptor) of Cys919 and the H31 atom (H-donor) of the ligand interacted by a hydrogen bond with a distance of 2.5 Å. Another hydrogen bond was detected between the HN atom (H-donor) of Asp1046 and the O12 atom (H-acceptor) of the ligand with a bond length of 2.2 Å. Additionally, ring A of the ligand interacted with Leu889 (alkyl hydrophobic, bond length 4.5 Å). The ring B with Leu889 (π–alkyl, bond length 5.3 Å), Lys868 (π–cation, bond length 3.5 Å), Val916 (π–alkyl, bond length 4.2 Å), ring C with Leu840 (π–alkyl, bond length 4.3 Å) and ring D with Leu840 (π–alkyl, bond length 5.2 Å), correspondingly, Figure 5H and Table 3 and Supplementary Figure S1.

## 3. Materials and Methods

### 3.1. Structure-Based Pharmacophore Generation

The structure-based pharmacophore method is one of the finest methods adapted in the field of computational drug discovery that critically probes into the features of the co-crystallised ligand. For the current study, the crystal structure of VEGFR-2 kinase domain (PDB code: 4AG8) in complex with axitinib was considered to generate the pharmacophore model. Correspondingly, the residues within 10 Å around the bound ligand were preferred to elucidate the key complementary features enabling the *Interaction Generation* module embedded with the Discovery Studio v4.5 (DS). Subsequently, the *Receptor-ligand Pharmacophore Generation* was employed to generate the pharmacophore models.

### 3.2. Pharmacophore Validation

Pharmacophore validation is one of the robust methods in assessing the ability of the selected pharmacophore model to retrieve active compounds. Accordingly, for the current study, a double validation method, such as the receiver operating characteristic (ROC) curve and the Güner-Henry approach (decoy set) approach was used. 

The ROC was executed along with the generation of the model with 18 active compounds and 27 inactive compounds, and the results were evaluated based upon the area under the curve (AUC). 

The Güner-Henry approach was conducted by instituting an external dataset (D:720) of experimentally known inhibitors derived from the same biological assay. The dataset consists of 24 compounds exhibiting an IC_50_ value of less than 100 nmol/L called the actives (A). The selected model was upgraded to screen the dataset utilizing the *Ligand Pharmacophore Mapping* tool accessible with the DS with the *FAST* algorithm. Furthermore, meticulous evaluation of the usability of the pharmacophore model was performed by calculating the enrichment factor (EF) and goodness of fit score (GF) enumerated by

(1) $$\mathrm{GF}=\left(\frac{\mathrm{Ha}}{4\mathrm{HtA}}\right)\left(3A+\mathrm{Ht}\right)X\;\left\{1-\frac{\mathrm{Ht}-\mathrm{Ha}}{D-A}\right\}\;$$

(2) $$\mathrm{EF}=\frac{\mathrm{Ha}\;X\;D}{\mathrm{Ht}\;X\;A}\;$$

### 3.3. Virtual Screening for the Retrieval of Drug-Like Compounds

The well-validated pharmacophore model was advanced to screen the chemical database to subsequently redeem the compounds that obey to pharmacophore features. Accordingly, the compounds from the Eximed (https://eximedlab.com/libraries.html), particularly the Natural Product Based Library (NPBL) and Natural Product Like Library (NPLL), were employed. The obtained compounds were determined for their drug-likeness and pharmacokinetics by absorption, distribution, metabolism and excretion—toxicity (ADMET) and Lipinski’s Rule of 5 (Ro5). The *ADMET Descriptors* available with the DS were recruited to assess the pharmacokinetic properties of the compounds, such as absorption, distribution, metabolism, excretion, and toxicity. The resultant compounds were processed to determine the physicochemical properties of the compounds by applying the Lipinski’s Rule of 5 (Ro5) available within the DS. The Ro5 predominantly governs the physicochemical properties of the compounds, i.e., when a compound has a molecular weight less than 500 kDa, and the compound’s lipophilicity (log*P*) and the number of hydrogen bond donors are less than 5, the number of hydrogen bond acceptor groups and rotatable bonds is less than 10, respectively.

### 3.4. Molecular Docking-Based Screening

Molecular docking is a method employed to determine the affinities between the protein and the screened compounds, thereby, elucidating the prospective binding mode of the ligands. For the current investigation, the CDOCKER protocol obtainable with the DS that implements on the CDOCKER algorithm was employed. This is a grid-based method that employs Chemistry at HARvard Macromolecular Mechanics (CHARMm) wherein the protein is held rigid while the ligands were allowed to flex. The results are evaluated based upon the -Cdocker interaction energy.

The protein for the current study is VEGFR-2 kinase domain in complex with axitinib, which was subsequently prepared using the *clean protein* module equipped with the DS. Furthermore, the missing residues and the hydrogen atoms were supplemented after removal of the water molecules and the bound ligands. The binding site of the protein was evaluated for all the atoms in vicinity of 10 Å residues around the co-crystallised ligand. 

The drug-like compounds obtained from the virtual screening steps were prepared by minimising them after removing the duplicates. They were escalated to molecular docking mechanism against the protein, allowing the generation of 50 conformations for each ligand and were subsequently clustered. The best binding pose was secured from the largest cluster representing the highest dock score rendered by the key residue interaction at the binding pocket. Furthermore, the quintessential binding modes were refined using the MD simulations employing GROningen MAchine for Chemical Simulations (GROMACS) v5.0.6.

### 3.5. Molecular Dynamics Simulation Studies 

Molecular dynamics (MD) simulation studies have been extensively employed to delineate on the molecular motion, predict the enzyme mechanism and further to comprehend the complex assemblies. The MD simulations additionally disseminate knowledge on the nature of the small molecules with its protein at the atomistic level [36,37]. For the current investigation, MD was employed to assess the stability of the target and the ligand complexes which could be analysed according to the radius of gyration (Rg), root mean square deviation (RMSD), and the potential energies recruiting GROMACS v5.0.6. The MD was launched with the ideal binding modes of the protein in complex with corresponding inhibitors from the molecular docking results utilising the all atom CHARMm27 force field [38] and retrieving the ligand topologies from SwissParam [39]. A dodecahedron water box [40] was built solvated with transferable intermolecular potential with 3 points (TIP3P) water model and were neutralised with counter ions. The steepest descent energy minimisation algorithm was used to relax the initial structures. Furthermore, the number of steps was confined to 10,000 using a minimisation force of less than 10,000 kJ/mol. Following this, a twofold equilibration was applied using a constant number of particles, volume, and temperature complex (NVT) and number of particles, pressure, and temperature (NPT), respectively. The first step of equilibration was conducted with a constant number of particles, volume, and temperature complex (NVT) at 300 K for 1 ns monitored by V-rescale thermostat. The second equilibration step was performed for number of particles, pressure, and temperature (NPT) ensemble for 1 ns controlling the pressure at 1 bar with Parrinello–Rahman barostat [41]. During the equilibration steps, the backbone of the protein was maintained rigid, while allowing the movement of the solvent molecules and the counter ions. The equilibrated ensembles were subjected to MD simulations conducted for 50 ns employing the LINear Constraint Solver (LINCS) and SETTLE [42,43] algorithm for bond constraints and geometry of water molecules. The particle mesh Ewald (PME) [44] method was used to calculate the long-range electrostatic interactions by defining a cut-off value of 9 Å and 14 Å for short-range interactions and van der Waals interactions, correspondingly. The obtained results were evaluated employing visual molecular dynamics (VMD) [45], GROMACS, and DS. 

## 4. Discussion

Small molecules that inhibit the protein kinases have been in increasing demand as an alternative therapy to mitigate the cancer metastasis. VEGFR-2 is a key player that is credited with angiogenesis and cancer growth [46,47]. The ATP binding pocket of VEGFR-2 acts as active site for small molecule inhibitors that are largely competitive inhibitors to the ATP [15,48]. Generally, the kinase inhibitors are of four different types; type 1 inhibitors bind to the active form of the kinases, while the type II interact with the inactive form of the kinases and bind with the residues located at the ATP binding site and the allosteric site. Type III inhibitors prefer to bind to the allosteric site, adjacent to the active site and the type IV inhibitors occupy the allosteric site away from the ATP binding site [49,50,51]. Furthermore, the residues Glu917 and Cys919 form the front pocket while the residues Glu885 and Asp1046 demonstrate the back pocket. The inhibitors binding with a back-to-front approach have been considered potential. Our earlier investigation retrieved only one compound that obeys this pattern of binding [36] and, therefore, we ventured to screen the natural compounds database in pursuit of obtaining more prospective candidate compounds. The NPBL resulted in 15 compounds, and NPLL put forth 11 compounds showing interactions with the key residues. A thorough investigation was conducted to retrieve the compounds with a back-to-front approach of binding redeeming three compounds from the NPBL and four compounds from NPLL, respectively. 

**Figure 6:**
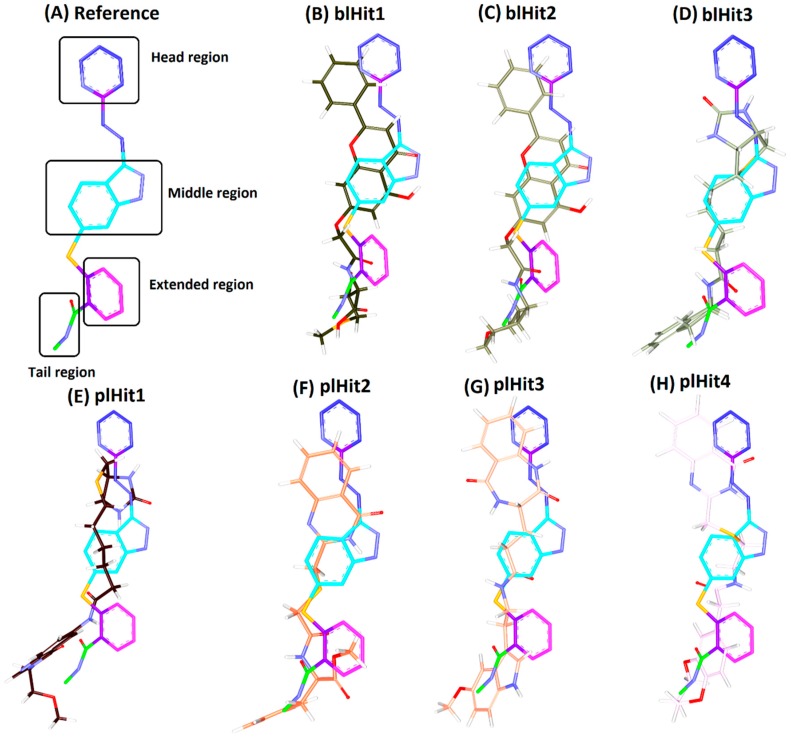
Elucidating the structural activity relationship (SAR). Different colours encoding the regions representing the reference compound. The identified compounds are represented by defined colours. (**A**) Different groups of the reference compound that occupy the active site; Figures (**B**–**H**) demonstrates the structural alignment of the identified compounds with the reference compound. (**B**) Alignment of blHit1 and the reference; (**C**) Overlay of reference and blHit2; (**D**) Alignment of reference and blHit3; (**E**) Alignment of reference and plHit1; (**F**) Alignment of reference and plHit2; (**G**) Alignment of reference and plHit3; (**H**) Alignment of reference and plHit4.

**Figure 7:**
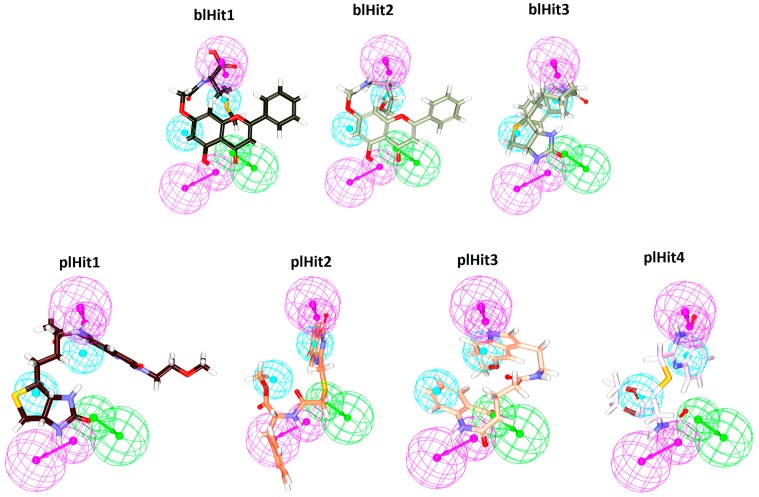
Alignment of the identified compounds with the pharmacophore. All the compounds represent the pharmacophore features.

To predict the potentiality of the identified compounds, a structure activity relationship (SAR) analysis was conducted. The reference compound was divided into four features, such as the head region, middle region, tail region and the extended region, Figure 6A. It was observed that all the identified compounds demonstrated a benzene ring at the head region that aligned perfectly with the reference compound, Figure 6. Introspecting the middle region, it was observed that only two compounds namely, blHit1 and blHit2 represented the ring structures at the middle region as was noticed with the reference compound. The remaining compounds demonstrated linear structures, Figure 6, that prompted a variety of bonds, Supplementary Figure S1, firmly locking the ligands at the binding pocket. Furthermore, the reference compound had a benzene ring at the extended region. The three hits, blHit1, blHit2 and blHit3, has an oxygen atom, while the plHit1 and plHit2 demonstrated a linear and extended linear chain. The plHit3 and plHit4 had demonstrated a ring complementing it, Figure 6. The tail region had a linear structure in all the compounds as was noticed in the reference compound, Figure 6. However, it was interesting to note that the plHit3 had a ring structure. These observations guide us to comprehend that the identified Hits represented the key features demonstrated by the reference compound and represented the pharmacophore features, Figure 7, and could, thereby, serve as an alternative anti-angiogenic therapeutics.

The identified compounds occupied the ATP binding site rendering crucial interactions with the key residues forming hydrogen bonds. Additionally, the candidate compounds formed several interactions with residues, such as Ile888, Leu889, Ile892, Val899, Leu1019 and Ile1044, identifying as type-II inhibitors extending towards the hydrophobic region. The significance of these residues has already been reported to enhance the binding affinity [52,53]. These results illuminate the potentiality of the identified compounds as alternative VEGFR-2 inhibitors and acting as initial structures/chemical spaces for further development. 

## 5. Conclusions

The current study exploits the binding features of cocrystal axitinib (herein regarded as reference to select the compounds from the database) to identify the candidate compounds that bind at the active site in a back-to-front approach. A systematic pharmacophore modelling and virtual screening processes were undertaken which led to the identification of seven compounds. These compounds generated a molecular dock score higher than the reference compound and prompted a higher number of van der Waals interactions. The MD studies showed that the compounds rendered stable RMSD results signifying the stability of the systems. These compounds additionally exhibited a higher number of hydrogen bond interactions than the reference compound and extending into the hydrophobic pocket. Taken together, our findings put forth seven candidate compounds, three from the natural compounds (NPBL) and four from the natural compound analogues (NPLL) as novel VEGFR-2 inhibitors that bind in a back-to-front approach.

## Figures and Tables

**Table 1:** Receptor-ligand based pharmacophore models and their distinct features.

Model No	Number of Features	Feature Set *	Selectivity
Model 1	5	HBD, HBD, HyP, HyP, HBA	9.84
Model 2	5	HBD, HyP, HyP, HBA, HBA	8.93
Model 3	5	HBD, HyP, HyP, HyP, HBA	8.93
Model 4	4	HBD, HBD, HyP, HBA	8.33
Model 5	4	HBD, HBD, HyP, HBA	8.33
Model 6	4	HBD, HBD, HyP, HyP	8.33
Model 7	5	HBA, HBA, HyP, HyP, HyP	8.01
Model 8	4	HBD, HBA, HyP, HyP	7.41
Model 9	4	HBD, HBA, HyP, HyP	7.41
Model 10	4	HBD, HBA, HyP, HyP	7.41

* HBD, hydrogen bond donor; HBA, hydrogen bond acceptor; HyP, hydrophobic.

**Table 2:** Different parameters computed through decoy set method.

S. No	Parameters	Values
1	Total number of molecules in database (D)	720
2	Total number of actives in database (A)	24
3	Total number of hit molecules from the database (Ht)	26
4	Total number of active molecules in hit list (Ha)	23
5	% Yield of actives (Ha/Ht)	88
6	% Ratio of actives [(Ha/A) × 100]	95.8
7	Enrichment factor (EF)	26.53
8	False negatives (A-Ha)	1
9	False positives (Ht-Ha)	3
10	Goodness of fit score (GF)	0.87

**Table 3:** Tabulation of different interactions prompted by protein-hit complex.

Compound Name	Hydrogen Bond Interactions < 3 Å	π–π/π–alkyl Interactions	van der Waals Interactions
Reference	Glu885:OE2-N82 (2.6) Glu917:O-N15 (2.8) Cys919:N-N14 (2.9) Asp1046:N-O81 (2.9)	Leu840, Val848, Ala866, Lys868, Cys1045, Phe1047	Val867, Leu889, Val899, Val914, Phe918, Lys920, Gly922
blHit1	Glu885:OE2-H40 (2.1) Glu917:O-H37 (1.9) Cys919:HN-O15 (1.9) Asp1046:O-H35 (2.0)	Leu840, Val848, Ala866, Leu1035, Cys1045	Ile888, Leu889, Ile892, Val899, Val914, Val916, Lys920, Gly922, Asn923, Thr926, His1026, Ile1044, Phe1047, Ala1050
blHit2	Glu917:O-H35 (1.9) Cys919:HN-O12 (1.8) Asp1046:O-H34 (1.9)	Leu840,Val848, Ala866, Leu889, Leu1035, Cys1045	Lys868, Glu885, Ile888, Ile892, Val899, Phe918, Lys920, Gly922, Val914, Asn923, Thr926, Ile1044, Ile1045, Phe1047
blHit3	Lys868:HZ3-O17 (1.9) Asp1046:O-H34 (2.1) Asn923:HN-O14 (2.8) Cys919:O-H27 (2.1)	His1026	Val848, Ala866, Glu885, Ile888, Leu889, Ile892, Val899, Val916, Phe918, Gly922, Leu1019, Leu1035, Ile1044, Phe1047
plHit1	Glu885:OE2-H41 (2.8) Cys919:HN-O19 (2.1) Cys919:O-H33 (2.6) Asp1046:HN-O21 (2.6)	Leu840, His1026, Asp1046	Gly841, Ala866, Leu889, His891, Val899, Val914, Val916, Glu917, Gly922, Asn923, Met1016, Leu1019, Arg1022, Cys1024, Ile1025, Arg1027, Leu1035, Cys1045, Phe1047, Ala1050,
plHit2	Cys919:HN-O11 (2.6) Asp1046:O-H32 (2.0)	Leu840,Val848, Ala866, Lys868, Leu889, Val916	Glu885, Ile892, Val899, Val914, Glu917, Phe918, Lys920, Gly922, Asn923, Leu1019, His1026, Leu1035, Ile1044, Phe1047
plHit3	Glu885:OE2-H34 (2.2) Cys919:O-H32 (2.4) Lys920:O-H32 (2.9) Asn923:HN-O14 (1.9) Asn923:HD21-O14 (1.9)	Leu840, Lys868, Leu889, Val916	Leu840, Val848, Ala866, Ile888, Ile892, Val899, Val914, Glu917, Phe918, Gly922, Leu1091, His1026, Leu1035, Ile1044, Cys1045, Phe1047, Asp1046
plHit4	Cys919:O-H31 (2.5) Asp1046:HN-O12 (2.2)	Leu840, Lys868, Leu889, Val916,	Val848, Ala866, Glu885, Ile888, Ile892, Val914, Glu917, Phe918, Lys920, Gly922, Thr926, Leu1035, Phe1047

## Supplementary Materials

The supplementary materials are available online at https://www.mdpi.com/2073-4409/8/3/269/s1.
