# Supplementary material for: Discovery of Small Molecules That Target Vascular Endothelial Growth Factor Receptor-2 Signalling Pathway Employing Molecular Modelling Studies
Source: Cells. 2019 Mar 21;8(3):269. doi: 10.3390/cells8030269 (PMC6468367; doi:10.3390/cells8030269)

Discovery of Small Molecules that Target Vascular Endothelial Growth Factor Receptor2 (VEGFR2) Signalling Pathway Employing Molecular Modelling Studies

Shailima Rampogu[1†], Ayoung Baek[1†], Chanin Park[1], Minky Son[1], Shraddha Parate[1], Saravanan Parameswaran[1], Yohan Park[2], Baji Shaik[3], Ju Hyun Kim[3], Seok Ju Park[4*] and Keun Woo Lee*

[1]Division of Life Sciences, Division of Applied Life Science (BK21 Plus), Plant Molecular Biology and Biotechnology Research Center (PMBBRC), Research Institute of Natural Science (RINS), Gyeongsang National University (GNU), 501 Jinju-daero, Jinju, 52828 Republic of Korea

[2]College of Pharmacy, Inje University, 197 Inje-ro, Gimhae, Gyeongnam 50834, Republic of Korea

[3]Department of Chemistry (BK 21 plus), Research Institute of Natural Science (RINS), Gyeongsang National University 52828, Jinju, Gyeongnam, Republic of Korea

[4]Department of Internal Medicine, College of Medicine, Busan Paik Hospital, Inje University, Gyeongnam, Republic of Korea

† Equal contribution

Supplementary Figure 1 . Detailed intermolecular interactions between the protein and the identified compounds in comparison with the reference compound.

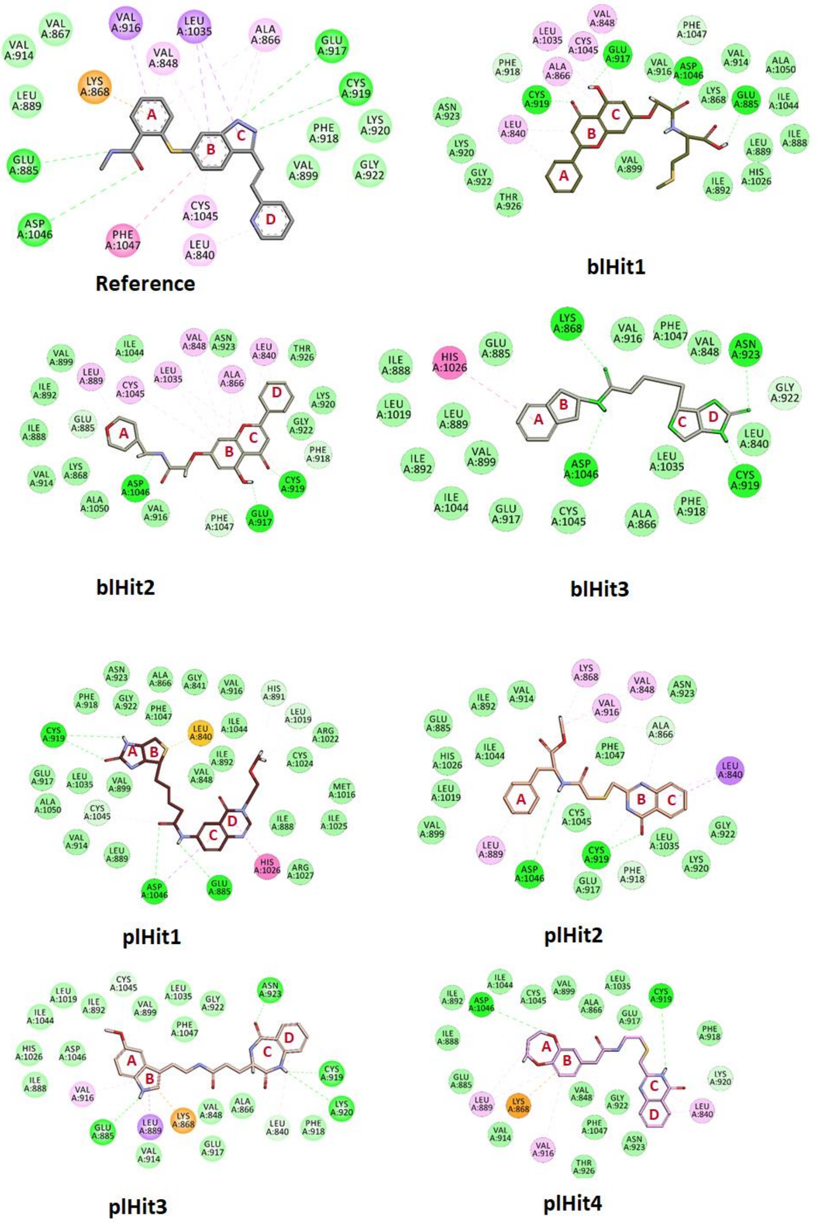

Supplement: Supplementary file 1 [file cells-08-00269-s001.pdf]
